# MMPs at Work: Deciphering Their Role in the Cellular Mechanisms of Orthodontic Tooth Movement

**DOI:** 10.3390/ijms27010542

**Published:** 2026-01-05

**Authors:** Mariana Ramos Patrão, Pedro Mariano Pereira, Jorge Caldeira, Madalena Salema-Oom

**Affiliations:** 1 Egas Moniz Center for Interdisciplinary Research (CiiEM), Egas Moniz School of Health and Science, 2829-511 Caparica, Portugal; pmarianop@egasmoniz.edu.pt (P.M.P.); jcaldeira@egasmoniz.edu.pt (J.C.); moom@egasmoniz.edu.pt (M.S.-O.); 2 Orthodontics Department, Instituto Universitário Egas Moniz, 2829-511 Caparica, Portugal; 3 Associated Laboratory for Green Chemistry (LAQV Requimte), Faculdade de Ciências e Tecnologia, Universidade Nova de Lisboa, 2829-516 Caparica, Portugal

**Keywords:** matrix metallopeptidases, periodontal ligament, orthodontic tooth movement, compression side, tension side

## Abstract

Matrix metallopeptidases (MMPs) are enzymes that, in balance with their inhibitors, play a vital role in extracellular matrix remodelling, particularly during orthodontic tooth movement (OTM). Despite growing interest, significant research is still required to fully comprehend the mechanisms and signalling pathways involved in periodontal ligament remodelling and OTM, particularly those mediated by MMPs. This review explores recent *in vitro* and *in vivo* evidence on how specific MMPs—namely, MMP-1, -2, -3, -8, -9, -12, -13, and -14—respond to compressive and tensile forces, regulate collagen degradation, and influence periodontal ligament fibroblast and osteoblast behaviour, ultimately shaping tissue resorption and formation. We also summarize the roles of periodontal ligament cells, hypoxia, the neurovascular and immune systems, and well-known molecules—including receptor activator of nuclear factor kappa β, receptor activator of nuclear factor kappa β ligand, osteoprotegerin, macrophage colony-stimulating factor, tumour necrosis factor α, transforming growth factor, and interleukins—in orchestrating these responses. Finally, we address the clinical relevance of these pathways, highlighting the potential for therapeutic strategies targeting MMPs activity. Overall, this review underscores the pivotal contribution of MMPs to extracellular matrix turnover and tissue adaptation during OTM and suggests that modulating the MMPs/tissue inhibitors of matrix metallopeptidase (TIMPs) balance may enhance orthodontic outcomes.

## 1. Introduction

Matrix metallopeptidases (MMPs) are a family of enzymes involved in the degradation and activation of extracellular matrix (ECM) components [1] during various physiological processes, including growth, haemostasis, tissue repair, and inflammatory and immune responses [2]. These endopeptidases are also critically involved in the remodelling of the ECM of the periodontal ligament (PDL), a key event in orthodontic tooth movement (OTM) [3]. The MMPs-mediated remodelling of the PDL plays a pivotal role in enabling the biological adaptation required for tooth displacement under mechanical forces.

OTM repositions teeth within the dental arches to correct malocclusions, prevent oral pathologies such as caries, gingivitis, and periodontitis, and enhance aesthetics [4,5]. Several models have been proposed to explain how orthodontic forces are converted into biological activity, as OTM reflects periodontium remodelling. Most theories initially focused on alveolar bone remodelling, such as the bone bending theory, proposed by Farrar, and the pressure–tension theory, advocated by Sandstedt, Oppenheim, and Schwarz [6]. Upon force application, the tooth shifts within the bone socket, creating two areas: a compression (resorption) side and a tension (apposition) side [4,7]. The biological electricity theory [8] and the piezoelectric theory [9] propose that the mechanical deformation of periodontium tissues, particularly the alveolar bone, generate electrical signals that modulate cell activity. However, more recent studies highlight the crucial role of the PDL in tooth movement [10,11,12]. The biphasic theory suggests that OTM involves an initial catabolic phase followed by an anabolic phase, with the PDL being the first structure to detect force and initiate the inflammatory cascade [13].

The physiological response of the PDL, its cells, and the molecules involved in the signalling pathways of OTM, particularly MMPs, remains partially unknown, with some studies reporting conflicting findings regarding their regulation and activity under compressive and tensile forces. Only by recognizing these mechanisms and identifying their chemical players in detail can we contribute to the development of novel therapeutic strategies to optimize orthodontic outcomes [4,14], enhance treatment success and efficiency, and minimize adverse effects such as root resorption and prevent relapse [15]. This review aims to provide a comprehensive and up-to-date overview of the MMPs underlying OTM, highlighting the key role of fibroblasts and osteoblasts. Accumulating evidence highlights MMPs as central regulators of ECM turnover, positioning them as promising targets to enhance orthodontic outcomes; however, their therapeutic relevance remains understudied.

### Search Strategy

The PubMed, Google Scholar, Scopus, and Cochrane Central Register of Controlled Trials databases, as well as leading journals in the field, were searched up to and including December 2025. The reference lists of the included and relevant articles were also searched manually. There were no limitations regarding the year of publication. English-language articles related to MMPs and OTM were included. Studies that did not meet these criteria after an initial abstract review, as well as case reports/series and expert opinion/consensus articles, were excluded.

## 2. Actors Involved in Periodontium Remodelling

The periodontium includes four different tissues—the gingiva, cementum, PDL, and alveolar bone—whose function is to provide insertion, support, protection, and lining to the tooth. The PDL is a fibrous connective tissue that connects the tooth (cementum) to the alveolar bone and is involved in important oral processes, such as sensory perception and dissipation of masticatory forces, tooth eruption, and, finally, OTM [16]. The PDL is constituted by a cellular component comprising fibroblasts, epithelial cells, and cells from the neurovascular and immune systems [17] and by an ECM. The interface with the alveolar bone is populated by osteoblasts and osteoclasts, and the interface with the cementum is mainly populated by cementoblasts [17]. The ECM is a component that provides structure to the PDL and is mainly made up of collagen type I organized into fibrils and, to a lesser extent, collagen type II, III, IV, V, VI, XI, XII, XIV, XV, and XVI, glycoproteins (e.g., fibronectin, periostin, and tenascin-N), proteoglycans (e.g., asporin, hyaluronic acid, and lumican), interstitial fluid, and lymphatic and blood vessels [16,18,19]. The ECM is also involved in the regulation of cellular activity [20,21], being essential for tissue growth and regeneration [20,21]. This regulation occurs through transmembrane proteins such as integrins and adhesion receptors, which link the ECM to the cell interior [22,23], and indirectly through the signalling and diffusion of growth factors and cytokines, which provide extracellular biomechanical signals to cells [20,21]. Fibronectin binds integrins, forming a bridge between ECM proteins (e.g., collagen) and actin microfilaments, facilitating intracellular signal transmission [22,24]. Integrin activation leads to structural changes exposing a cytoplasmic domain that enhances focal adhesion kinase (FAK) activity, triggering cytoskeletal rearrangements, cytokine release, and alterations in gene expression [21].

Following force application, the tooth moves within the alveolus, deforming the PDL and, consequently, the surrounding bone [25]. This deformation affects the viscoelastic ECM and the nerve endings and alters the vascular and interstitial fluid flow [13,26]. On the resorption side, the PDL space narrows, and collagen fibres, blood vessels, and nerve endings compress, leading to a decrease in blood flow and local hypoxia [4,7]. On the apposition side, the opposite occurs—the PDL space widens, collagen fibres stretch, and blood vessels dilate, increasing blood flow [4,7]. These changes trigger an aseptic inflammatory cascade regulated by chemical mediators [25,27,28]. These topics are discussed below.

### 2.1. Main Cells

Fibroblasts, osteoblasts, osteocytes, and osteoclasts are the key regulators of OTM [15].

#### 2.1.1. Fibroblasts

Fibroblasts are the primary cellular component of the PDL, representing 50-60% of its cells [29,30]. They are responsible for the production of collagen and other ECM constituents, ensuring PDL turnover and structural integrity [31,32]. As primary mechanical sensors of the PDL, fibroblasts detect orthodontic forces and exhibit high turnover rates during OTM [15]. They secrete factors that influence periodontium remodelling and regulate osteoblast and osteoclast activity [32,33]. Additionally, fibroblasts are involved in innate immune system regulation [31,32].

#### 2.1.2. Osteoblasts

Osteoblasts are responsible for bone induction and formation and also contribute to bone resorption [30,34,35]. They originate from mesenchymal stem cells that differentiate into osteoblast precursors, immature osteoblasts, and, finally, mature osteoblasts [36,37]. Osteoblasts initially secrete osteoid [38], a non-mineralized bone tissue composed of type I collagen, non-collagenous proteins, and proteoglycans [39,40]. Subsequently, they mineralize the matrix by depositing hydroxyapatite crystals [41]. At the end of their lifespan, osteoblasts undergo apoptosis or give rise to osteocytes or bone lining cells [29,34,42].

#### 2.1.3. Osteocytes

Osteocytes derive from osteoblasts and are the most abundant bone cells [43]. They are embedded in the bone, within mineralized lacunae where the circulating fluid supplies oxygen and nutrients [44]. The position occupied by these cells makes them perfect mechanoreceptors [44], capable of sensing mechanical stimuli (e.g., changes in canalicular fluid) and identifying physiological bone variations [40]. Upon force application, osteocytes interact with other cells [40] through long dendrites branching out across the bone matrix and establish a complex intercellular communication network that regulates osteoblast and osteoclast activity [45].

#### 2.1.4. Osteoclasts

Osteoclasts are responsible for bone dissolution [46]. They originate from the differentiation of haematopoietic stem cells into colony-forming unit-monocytes, followed by monocytes, mononuclear osteoclasts, and, finally, multinuclear osteoclasts [39,47,48].

### 2.2. Systemic Contributors

In addition to the cells considered in Section 2.1, other systems play an important role in OTM.

#### 2.2.1. Immune System

The application of orthodontic force triggers an inflammatory and immunological response. Neutrophils are the first cells to migrate to the site, followed by leukocytes and monocytes/macrophages [49]. On the resorption side, pro-inflammatory M1-type macrophages release interleukins (IL)-1 and -6 and tumour necrosis factor-alpha (TNF-α), which promote osteoclast differentiation and, consequently, periodontium resorption [50]. Compression-activated T lymphocytes release receptor activator of nuclear factor kappa-B ligand (RANKL) [51], a primary driver of osteoclastogenesis, which binds to the receptor activator of nuclear factor kappa-B (RANK) present in osteoclast precursor cells [49]. T lymphocytes also release TNF-α and gamma interferon (IFN-γ), responsible for M1-type macrophage polarization [52]. Conversely, on the apposition side, anti-inflammatory M2-type macrophages produce IL-10 and transforming growth factor beta (TGF-β), which signal the cessation of resorption and the onset of periodontium formation [53,54]. T cells influence B cells to release osteoprotegerin (OPG), which binds to RANKL, preventing its interaction with RANK and thereby suppressing bone resorption [52].

#### 2.2.2. Neurovascular System

The PDL is irrigated by blood vessels and is highly innervated by mechanical and nociceptive receptors [55]. On the resorption side, PDL cells express hypoxia-induced transcription factor 1 alpha (HIF-1α), which positively regulates the expression of genes: *RANKL* in fibroblasts [56]; vascular endothelial growth factor (*VEGF*), which upregulates *RANKL* in fibroblasts and osteoblasts, promoting osteoclastogenesis [56,57,58]; and *MMPs*, involved in extracellular proteolysis [59,60]. Additionally, stimulated nerve endings release the neuropeptides norepinephrine (NE) and substance P (SP), promoting RANKL and inflammatory marker expression in osteoblasts [61,62,63]. SP also contributes to this process by activating the NF-kB signalling pathway [64]. On the apposition side, VEGF’s actions are also felt in angiogenesis, promoting the migration of osteoblast precursor cells and enhancing vascular permeability [65,66]. This ensures a correct supply of oxygen and nutrients to the cells [65,66]. Neuropeptides such as vasoactive intestinal polypeptide (VIP), calcitonin gene-related peptide (CGRP), and SP are released [63,67,68], promoting VEGF production [67,69,70,71]. VIP and SP also enhance type I collagen synthesis, *Runx-2* transcription factor (essential for osteoblastogenesis), and alkaline phosphatase expression (essential for the mineralization process) [40,43,64,72]. In addition, SP promotes endothelial cell chemotaxis to the PDL [69]. Finally, CGRP contributes to M2-type macrophage polarization and stimulates the release of anti-inflammatory cytokines and growth factors, supporting fibroblast and osteoblast proliferation and collagen/bone formation [73,74]. Furthermore, VIP and CGRP potentiate OPG production by osteoblasts, blocking osteoclastogenesis [75,76].

### 2.3. MMPs

The most drastic changes in periodontium remodelling occur in the PDL, with the degradation of the ECM [77]. Fibroblasts are the first cells to be activated in response to force application, secreting molecules such as MMPs [78]. MMPs are endopeptidases responsible for the turnover of collagen, gelatine, glycoproteins and proteoglycans, and elastin, as well as other MMPs and cytokines, membrane receptors, and growth factors [2,79,80]. These proteolytic enzymes belong to the metzincin superfamily, since their function is dependent on ions such as calcium (Ca^2+^) and zinc (Zn^2+^) [81]. To date, the MMPs family includes at least 23 different peptidases in human tissues, classified into six distinct classes based on the organization of their specific domains and substrates (collagenases, gelatinases, stromelysins, matrilysins, membrane MMPs, and other MMPs) or grouped based on their cellular location (secreted MMPs and MMPs anchored to the cell membrane) [82]. Despite having different substrates, MMPs share similar characteristics [82]. With few exceptions, MMPs are made up of the pro(peptide) domain, the catalytic domain, the hemopexin-like c-terminal domain, a linker (which joins the last two domains, ensuring stability and flexibility), and a signal peptide, which, when cleaved, gives rise to prometallopeptidases [80,81].

#### Secretion and Activation of MMPs

MMPs are produced by multiple cells, including fibroblasts and osteoblasts [83,84]. Successful PDL homeostasis depends upon the regulation of MMPs activity. This can be achieved at four levels: (1) gene expression, (2) compartmentalization, (3) activation of proenzymes, and (4) inhibition [85]. These processes are influenced by growth factors, cytokines, proteases, and even other MMPs, as well as by endogenous and synthetic MMPs inhibitors [1,86]. Endogenous MMPs inhibitors can be classified as specific and nonspecific, with the former being known as tissue inhibitors of matrix metallopeptidases (TIMPs) [1,81].

**Figure 1:**
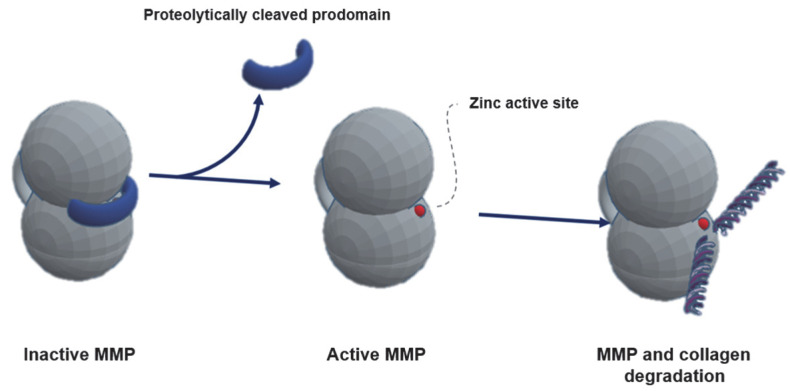
Proteolytic activation of matrix metallopeptidases (MMPs) with prodomain removal, exposing zinc catalytic site capable of collagen and other molecule cleavage.

Initially secreted as pre-prometallopeptidases [1], they become inactive zymogens (prometallopeptidases) after the N-terminal signal peptide is cleaved [79]. The prodomain is removed, either by proteolytic cleavage or structural alteration, leading to a stepwise activation of the MMPs zymogens [87]. Firstly, a susceptible zone of the prodomain, the bait region, is cleaved, rendering the catalytic zone accessible. Secondly, a range of enzymes (including other MMPs) may cleave and remove the final part of the prodomain [88]. The MMPs acquire their active form, thereby becoming capable of performing catalytic functions [80] (Figure 1).

It has been demonstrated that plasmin, in conjunction with MMP-3, has the capacity to activate MMP-1 [89,90]. In addition to MMP-3, MMP-10 can activate MMP-1, -8, and -9 but not MMP-2 or -3 [91,92]. MMP-14, an enzyme linked to fibroblast cell membranes, activates MMP-8 [93] and, in conjunction with plasmin and MMP-3, MMP-13 [94,95,96]. MMP-13 is self-activated by proteolysis [97]. Furthermore, MMP-14 has been observed to form a complex with TIMP-2, resulting in its capture and the release of active MMP-2 [98], which is also able to activate MMP-13 [94]. Consequently, active MMPs can activate gelatinases through the same process of proteolysis. MMP-1 has been shown to stimulate the activation of MMP-9 in a cyclooxygenase-2 (COX-2)-dependent manner [99]. In addition to COX-2, MMP-3 (directly) [100] and MMP-13 [101], as well as plasmin [102], also activate MMP-9, which, in turn, contributes to the activation of MMP-13 [101]. Table 1 illustrates the activation processes of the MMPs discussed.

The activity of MMPs is influenced by other molecules whose expression is altered on the resorption side too. For instance, plasmin and TNF-α, produced by fibroblasts and immune cells, have been shown to specifically enhance the expression of MMP-1 [103], -3 [95], -9 [104], and -13 [95]. Similarly, TGF-β, released by fibroblasts and osteoblasts, has been shown to upregulate the expression of MMP-2 and -9 [105]; and IL-1α has been shown to upregulate the expression of MMP-1 and -3 and to downregulate the expression of TIMP-2 [106,107]. Additionally, the hypoxic conditions have been observed to induce the over-expression of MMP-1 [108], -3 [109], -9 [110], -13 [111], and -14 [112]. Collectively, these observations reveal a complex regulatory interplay of several MMPs for tissue adaptation to mechanical stimulus. Once activated, it is the balance between MMPs and TIMPs that determines the turnover of the PDL [113], since TIMPs are negative regulators of MMPs functions [114].

TIMPs are produced by fibroblasts and are classified into four types—TIMP-1, -2, -3, and -4—which bind to the active zone of MMPs [115]. Although TIMPs are described as nonspecific inhibitors, there are differences in their activity [1]. For example, TIMP-1 has a ten-fold higher affinity for MMP-3 than for MMP-10 [116]. It is also described as inducible with a stronger inhibitor of MMP-1, -2, -3, and -9, while TIMP-2 is constitutive with a higher affinity for MMP-2 [80]. Although TIMPs are also present on the compression side (at lower levels, favouring resorptive processes), their higher levels on the tension side promote anabolic activity [31]. In fact, the decreased MMPs activity observed on the tension side appears to be due to TIMPs-mediated inhibition rather than decreased MMPs genetic expression [23,31,117,118,119,120,121,122].

## 3. Role of MMPs in Orthodontic Tooth Movement

**Figure 2:**
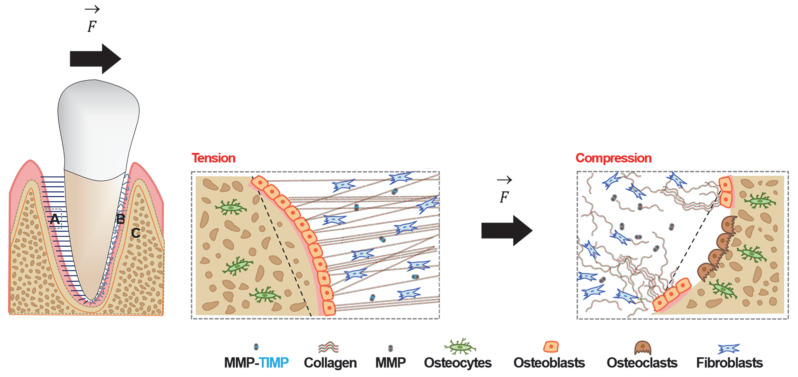
Schematic view of the bone–periodontal ligament (PDL)–tooth complex under orthodontic force ($\vec{F}$): (A) anabolic phase with stretched PDL fibres on tension side; (B) catabolic phase with compressed PDL fibres and bone resorption on compression side; (C) alveolar bone.

Evidence of MMPs expression in response to mechanical stimuli in OTM comes from both *in vitro* and *in vivo* studies [123]. *In vitro* studies use compression forces to simulate what may be occurring on the resorption side, while stretching forces tend to simulate the apposition side during OTM. *In vivo* studies use gingival crevicular fluid (GCF) or saliva to evaluate MMPs levels. The biological events that take place on either side of the tooth during OTM are illustrated in Figure 2.

### 3.1. MMPs on the Resorption Side

#### 3.1.1. *In Vitro* Evidence

- *MMPs* changes in PDL cells

In human PDL fibroblasts, stimulation by compression has been shown to result in the upregulation of MMP-1 [77,124,125,126], -2 [127], -3 [124], and -10 [124] for both mRNA and protein production. A contradictory result related to MMP-2 was found in one study, which showed a decrease in MMP-2 activity [128]. No alterations were identified in TIMP-1 or -2 [125], nor in MMP-7, -8, -9, -13, or -16 [126]. In contrast, heightened *MMP8* and *13* gene expression in PDL fibroblasts subjected to static compressive forces were observed in two studies [129,130]. The results with compressed PDL fibroblasts point to an effective role of collagenase MMP-1, gelatinase MMP-2, and stromelysins MMP-3 and MMP-10.

- MMPs changes in bone cells

Mechanical stimulation can upregulate the MMP-3 expression in osteoblasts, potentially through the p38 MAPK pathway [131]. Compressive stress has also been shown to significantly increase *MMP1*, *2*, *13*, and *14* gene and protein expression in comparison to control cells [132,133]. No alterations in MMP-8 protein synthesis were observed [130].

#### 3.1.2. *In Vivo* Evidence

*In vivo* studies have produced divergent results. Under physiological conditions, the basal expression of MMPs and the specific inhibition by TIMPs maintain MMPs activity balance [134]. However, when compressive forces are applied during orthodontic treatment and the aseptic inflammatory process is triggered, the expression of MMP-1 [118,135,136], -3 [137], -8 [138,139], and -13 [137,140,141] becomes upregulated. Still, in a study over a one-month period, MMP-1 was not detected [142]. The same study observed an increase in MMP-8 levels after four to eight hours of force application [142], and these levels seem to remain elevated after three months of fixed appliance use [141,143]. In contrast, a recent study reported no significant changes in MMP-8 levels [144]. The levels of the gelatinases MMP-2 and MMP-9 have been shown to increase in GCF collected from the compression sides [118,136,137,139,145], with MMP-9 showing a statistically significant rise after four hours of force application [146]. Interestingly, a comprehensive study analysing the MMP-1, -2, -3, -7, -8, -9, -10, -12, and -13 concentrations in saliva revealed that only MMP-8 and -9 underwent significant increases one hour after orthodontic appliance activation [147]. Furthermore, the MMP-8, -9, and -12 levels correlated with the extent of tooth movement in patients who underwent extractions [147].

There seems to be more evidence for the increased expression and, eventually, increased activity of MMP-1, -2, -3, -8, -9, -10, and -13 on the compression side, confirmed by some *in vitro* and *in vivo* studies. MMP-8 levels found *in vivo* appear to be produced by periodontal fibroblasts rather than osteoblasts [130].

### 3.2. MMPs on the Apposition Side

#### 3.2.1. *In Vitro* Evidence

- MMPs changes in PDL cells

When tensile forces were applied to PDL fibroblasts, the same inconsistent results observed with compressive forces were found. While the most frequently reported MMPs and TIMPs are MMP-1 and -2 and TIMP-1 and -2, it is important to note that the different protocols used to induce tensile stretch may contribute to different observations. Specifically, MMP-1 was observed to be either upregulated [117,120] or unchanged [127,148,149] in some studies while it was downregulated in others [150]. In the case of MMP-2, it was shown to increase [117,127,151] or be unchanged [148,151]. Chen et al. [151] observed that high-level stretching (up to 10% elongation) increased MMP-2 expression over 24 to 48 h, while for moderate elongation stress, no changes were detected. A similar dependence on the stretch level was observed for MMP-12 and -13 [23,122]. The rapid upregulation of these genes was shown to occur within six hours of moderate force stimulation via MAP-kinase (p42/44 and p38) activation [23,122]. Interestingly, despite this increase, the expression of TIMP-1, -2, and -3 remained unchanged [120,122]. However, high levels of elongation did not result in an increase in the same MMPs [149]. It is evident that the involvement of specific MMPs is not only related to their expression but also to their activation and inhibition. The natural inhibitors TIMP-1 and -2, which are known to be effective against several MMPs [1], have been shown to be upregulated in most studies independent of the level of the stimulus [117,119,148,150,151]. This increased expression may reflect their prominent role in balancing the active MMPs in the tissues undergoing ECM remodelling. For instance, MMP-8 showed a modest increase in stretched fibroblasts, but its activity appears to be regulated by a parallel increase in TIMP-1, resulting in a TIMP-1/MMP-8 ratio that favours ECM preservation rather than degradation [119]. The presence of gelatinase MMP-9, which has been found to be associated with numerous pathological processes, was not detected, and in another study, its levels persisted the same [117,149]. Similarly, the *MMP-14* mRNA expression in fibroblasts subjected to mechanical tension remained unchanged [117].

- MMPs changes in bone cells

In mechanically stretched osteoblasts, decreased MMP-2 activity alongside increased MMP-9 expression was found [152]. MMP-13 upregulation was also found in osteoblasts under tensile forces [121]. However, this increase was counterbalanced by TIMP-1 upregulation, limiting MMP-13 proteolytic activity [121].

#### 3.2.2. *In Vivo* Evidence

*In vivo* studies using GCF from the apposition side to evaluate changes in MMPs levels have variable results. In clinical studies, MMP-1 appears to be the most frequently evaluated, with additional attention given to MMP-2, -3, -8, -9, -12, and -13. *In vivo* MMP-1 and -2 levels rise [135] within the first hour of force application, returning to baseline after eight hours [118]. Although some studies have shown an increase in MMP-1, they found similar variations between the compression and tension sides [31,135]. The same comparable levels in the GCF from the two opposite sides of the tooth were observed for other MMPs, such as -2, -3, -7, -8, -12, and -13, that were comparable to the control sides too [31,153]. However, in another study, MMP-3 and -13 presented increased expression on the apposition side [137]. As mentioned for MMP-1 and -2, the gelatinase MMP-9 showed a significant rise at 4 h and 7 days upon application of an orthodontic appliance on both sides [135,137,146]. MMP-12 has been associated with angiogenesis on the apposition side since its expression increases significantly in regions adjacent to type IV collagen under endothelial cells [122]. This suggests a role in vascular remodelling and neovascularization [122].

Apposition-side activity appears more focused on ECM preservation rather than degradation, mediated by high TIMPs expression, minimal MMP-9 activity, and balanced collagen remodelling [135,146].

The variations in the main MMPs cited in the *in vitro* and *in vivo* studies are summarized in Table 2 and Supplementary Tables S1 and S2. The results described in the *in vivo* studies are inconsistent and somewhat divergent from the *in vitro* observations, precluding a firm conclusion about which MMPs are likely to be responsive to the forces. A multitude of factors may be hypothesized as contributors to the observed variations. *In vitro* studies show considerable methodological heterogeneity, primarily using primary fibroblasts subjected to compressive or tensile forces via centrifugation, static weights, or tension–compression devices. In addition to the type of force applied, variability in the force magnitude (1–20% elongation), loading mode (static or cyclic), or duration of force application poses a significant challenge when conducting such comparisons. Furthermore, most studies analysed only a selected number of MMPs or TIMPs, with MMP-1 and MMP-2 being the most consistently reported as having increased. Another important limitation of *in vitro* models lies with the use of monolayer cultures involving a single cell type, which fails to replicate the complexity of the PDL microenvironment. A final confounding factor concerns the nature of the variable measured. Genetic methodologies demonstrate variations in gene expression, and most immunoassays quantitate only MMPs proteins. However, it should be noted that MMPs activity is conditional on either enzyme activation or inhibition. The findings derived from *in vivo* studies are further restricted by variations in GCF collection, including inconsistent specification of the resorption and apposition sides, as well as potential molecule diffusion over time. Additional limitations arise from varying sampling times, which can range from minutes to months and may comprise acute and chronic effects of force application. Notably, GCF collection comprises a complex mixture of all factors and enzymes produced by all the cells at a given time point. This includes not only fibroblast and osteoblast cells but also endothelial cells and cells from the immune system. Despite these differences, the majority of the studies commonly report increased MMP-1, -2, -3, -9, and -13 during resorption. These acknowledged constraints observed in the *in vitro* and *in vivo* studies limit the translation of preclinical findings into clinical practice. Future research should prioritize the development of (1) advanced *in vitro* systems that better simulate the PDL characteristics, incorporating tridimensionality cultures with one or multiple cell types and ECM components; (2) well-controlled and longitudinal *in vivo* study designs that accurately reflect the temporal and biomechanical complexity of OTM. Greater emphasis should be placed on the use of standardized protocols that distinctly collect GCF from both sides of the OTM (resorption and apposition). Integrating *in vitro* models with *in vivo* clinical studies is critical to comprehend the biological aspects of OTM and clarify the therapeutic potential and safety of modulating *MMPs* activity in orthodontic treatment.

## 4. PDL (And Bone) Remodelling

### 4.1. Resorption Side/ECM Degradation

The collagenases (see Table 2 in Section 3.2) may initiate the degradation of the PDL, since, together with MMP-14, they are capable of degrading type I (and III) collagen, its main constituent [138,154]. Active MMP-1, -8, and -13 have been shown to bind to specific sites on the collagen chains, unwind, and then hydrolyse the triple helix, producing collagen fragments that are 25% and 75% longer [155]. The now-denatured collagen chains (gelatine) are separated and become susceptible to the gelatinases MMP-2 and -9, which are also secreted by fibroblasts and osteoblasts [156,157]. Collagenases degrade other ECM proteins, including fibronectin, laminin, and tenascin [97]. Gelatinases, in addition to their role in the degradation of gelatine, have been observed to hydrolyse type IV collagen [158], elastin [159], and fibronectin and laminin [158]. The stromelysins MMP-3 and -10, besides their role in MMPs activation, contribute to the degradation of type IV collagen, fibronectin, laminin, and proteoglycans [160,161]. These events result in the resorption of the PDL and the detachment of PDL collagen fibres from the bone, which is followed by the activation of osteoclasts and bone resorption events [25,130,162,163].

After being activated by a compressive force, fibroblasts and osteoblasts were shown to upregulate COX-2 expression, stimulating prostaglandin E_2_ (PGE_2_) production [164], thereby enhancing RANKL expression [164,165] and promoting osteoclast precursor differentiation into osteoclasts [29]. Osteoclastogenesis is further promoted by additional molecules, including VEGF, macrophage colony-stimulating factor (M-CSF), and TNF-α [166,167]. TNF-α induces RANKL expression in osteocytes and, together with the production of PGE_2_ and M-CSF by these cells, enhances bone resorption [166,168]. Osteocytes also produce sclerostin, which inhibits WNT signalling, suppressing OPG expression and osteoblast differentiation [169,170]. The RANKL/OPG ratio increases on the resorption side due to RANKL production and OPG suppression [171]. Pro-inflammatory cytokines IL-1, -6, and -8, released by fibroblasts and immune cells, amplify these effects by upregulating PGE_2_ and TNF-α [163,172]. In addition to being directly implicated in the degradation of the ECM, active MMPs have also been observed to regulate molecules involved in bone resorption: MMP-3 activates pro-IL-1β [95]; MMP-2, -3, and -9 cleave and activate osteopontin (OPN) [173]. After osteoid (non-mineralized) tissue degradation by the action of MMP-1 [174] and -13 [175], which are produced by osteoblasts [172,176], differentiated osteoclasts adhere to the now-exposed bone matrix via OPN, reorganizing their cytoskeleton to form resorption compartments [34,35,41]. Organic bone matrix degradation through activated osteoclasts is mediated by MMP-9, -12, and -13, cathepsin K, and tartrate-resistant acid phosphatase, while mineral dissolution occurs through proton secretion, leading to acidification [37,41,48,175] and the release of collagen type I fragments, Ca^2+^, and phosphate (PO_4_^3−^) into the bloodstream [47].

Tissue (PDL and bone) resorption on the resorption side creates the necessary space for tooth displacement and is a prerequisite for the beginning of tissue formation.

### 4.2. Apposition Side/Collagen Synthesis

Unlike the PDL degradation phenomenon that occurs on the compression side, the tension side is marked by the cellular activation of fibroblasts that favour synthesis phenomena. New collagen fibres and blood vessels are produced, which are responsible for PDL reorganization in the new tooth position.

When tensile forces deform fibroblasts, calcium channels are activated, triggering the MAPK signalling pathway, which positively regulates the transcription of c-fos [30,32,120]. This transcription factor binds to other factors forming the AP-1 complex, modulating gene expression in fibroblasts, namely, the expression of MMP-1 and COL-1, the predominant collagen type produced following mechanical stimulation [30,120]. Growth factors such as TGF-β [177], platelet-derived growth factor (PDGF) [178], connective tissue growth factor (CTGF) [179], fibroblast growth factor (FGF), and insulin-like growth factor (IGF-1) [180] activate intracellular signalling cascades in fibroblasts that also enhance the expression of collagen precursor molecules (procollagen), enzymes, and co-factors involved in collagen synthesis [177,180]. Collagen synthesis begins with DNA transcription in the nuclei of activated fibroblasts and osteoblasts [181]. The resulting procollagen molecules are secreted into the extracellular space, where they spontaneously aggregate and form mature collagen molecules [181]. The enzyme prolyl hydroxylase is essential for stabilizing the collagen triple helix—an oxygen, iron, and ascorbic acid-dependent process [182]—and for the formation of collagen fibrils [181]. Finally, these fibrils form covalent cross-links, resulting in collagen fibres with high resistance to mechanical loading [183]. Under PDGF induction, fibroblasts secrete fibronectin, involved in chemotaxis, proliferation, and adhesion of fibroblasts to the ECM [178]. Fibronectin also binds to collagen and proteoglycans present in the matrix, contributing to collagen fibre deposition and, consequently, ECM reorganization [184,185,186]. Furthermore, activated fibroblasts also produce periostin, a protein that is involved in collagen fibrillogenesis [115,187] and bone sialoproteins (BSP), a protein essential for PDL organization and attachment that promotes osteoblast differentiation [188]. Another molecule involved in PDL regeneration is VEGF, due to its angiogenesis actions (explained above in Section 2.2). These molecular mechanisms, along with mechanical stimuli such as collagen fibre stretching and alterations in fluid flow, contribute to bone formation during OTM [189].

Bone formation depends on osteoblast differentiation and the mineral regulation of ions such as Ca^2+^ and PO_4_^3−^, which are essential for the formation of hydroxyapatite crystals. Osteocytes detect changes in fluid caused by tension forces [40,44]. Calcium channels located on the surfaces of these cells are activated and an intracellular cascade is triggered, culminating in the production of molecules such as FGF-23, which plays a role in phosphate homeostasis [190], as well as nitric oxide and IGF-1, involved in the differentiation of osteoblasts [191,192]. Other factors involved in the differentiation of these cells include PDGF and bone morphogenetic proteins (BMPs), which are known for their great osteoinductive capacity [1,193,194,195]. BMPs belong to the TGF-β superfamily and positively regulate the Runx-2 transcription factor [196]. Concurrently with the synthesis of these osteoblast factors, the production of sclerostin by osteocytes decreases and, consequently, the (canonical) WNT signalling pathway is no longer inhibited [197]. Following the activation of osteoblasts, these cells are primarily responsible for the process of bone mineralization through the secretion of the osteoid matrix and its calcification [198]. Osteoblasts initially synthesize osteocalcin, osteonectin, type I collagen, proteoglycans, and bone sialoproteins, which are key components of osteoid tissue [199,200,201]. Osteocalcin contributes to organized bone growth, parallel to collagen fibrils [202]. OPG, produced by osteoblasts, inhibits osteoclast differentiation [47,203], whereas osteonectin, also secreted by PDL fibroblasts, facilitates type I collagen production and deposition [204]. In a subsequent phase, osteoblast-derived vesicles bind to the newly formed organic matrix, initiating mineralization [41]. Negatively charged ECM molecules, such as proteoglycans, retain Ca^2+^ ions, which accumulate into these vesicles [41]. Concurrently, osteoblasts produce alkaline phosphatase, an enzyme located on the outer cell membrane, which releases PO_4_^3−^ ions into the vesicles [205]. There, Ca^2+^ and PO_4_^3−^ precipitate as hydroxyapatite crystals [40,206]. BSPs produced by fibroblasts are implicated in the initiation of this hydroxyapatite formation [188]. Crystals are then deposited along adjacent collagen fibres, ensuring the proper orientation of the apatite mineral during its growth [46].

### 4.3. Pathological Conditions

It is important to note that when heavy/inadequate orthodontic forces are acting on the tooth for a certain period, a process called hyalinization may occur [207]. Hyalinization is a process of sterile necrosis characterized by the presence of dead cells in the PDL and adjacent bone due to a strong reduction in the oxygen and nutrient supply [208]. The cells present in the hyaline zones are unable to differentiate, preventing the process of remodelling until this tissue is eliminated [209]. The presence of extensive hyaline regions hinders their elimination and can result in a delay in OTM [210]. External apical root resorption is a complication that can arise from the removal of hyaline tissue when the cementoblasts in the vicinity of necrotic zones of the PDL undergo apoptosis, thereby allowing the odontoclasts to resorb the cementum and dentin, resulting in root shortening [211]. MMP-1, -2, and -9 and cathepsin K seem to be involved in this process as well [139,174,212].

## 5. Clinical Implications

Knowledge of the pathways leading to tooth movement is extremely important, as it helps orthodontists achieve better results. The use of gene therapy and the local administration of biomodulators during orthodontic treatment can solve problems such as lack of anchorage or unwanted relapse and can be used to obtain faster results and decrease root resorption, improving the stability of orthodontic treatment [14].

Synthetic MMPs inhibitors have been proposed as pharmacological targets for delaying OTM and reducing post-treatment relapse, as they decrease the activity of MMPs related to collagen renewal and the removal of hyalinized areas of the PDL [213,214]. Examples of these inhibitors include anti-tumour drugs such as prinomastat, which inhibits various MMPs, such as MMP-1, -2, -3, -7, -9, and -14; ilomastat, which inhibits various MMPs, including MMP-2 and -9; marimastat, which inhibits MMP-1, -2, -3, -7, -9, and -12; tanomastat, which inhibits MMP-2, -3, -9, and -13; metastat, which inhibits MMP-1, -2, -8, -9, and -13; and D2163, which inhibits MMP-1, -2, and -9 [213,215,216]. Other substances include disulfiram, which has been shown to selectively inhibit MMP-2 and -9 [217], while XL784 is recognized as a potent inhibitor of MMP-2 and -13 [218]. Doxycycline hydrochloride, a tetracycline antibiotic, exerts broad-spectrum inhibitory effects on MMPs activity, notably affecting MMP-2 and -9 production [216,219]. Similarly, statins exhibit the non-selective inhibition of MMPs, particularly affecting MMP-1, -2, -3, and -9 secretion [220]. Other drugs such as alendronate [221] and acetylsalicylic acid [222] inhibit MMP-2. However, most of the evidence supporting the use of synthetic MMPs inhibitors is derived from animal models or clinical contexts unrelated to orthodontics, such as oncology or other medical conditions. Importantly, one of the diseases in which the clinical relevance of synthetic MMPs inhibitors has been established is periodontitis [216,223]. In this context, doses of sub-antimicrobial doxycycline (Periostat), the only drug approved for periodontitis treatment, have demonstrated efficacy in inhibiting MMPs gene expression and proteolytic activity [216]. A regimen of 20 mg administered twice daily for periods of up to nine months has demonstrated a favourable safety profile in long-term clinical use, as this low-dose formulation does not exert antimicrobial effects or induce antibiotic-related adverse reactions [223]. Although supporting the translational potential of synthetic MMPs inhibition within the oral environment, comparable evidence regarding safe local or systemic dosages, long-term safety profiles, and specific clinical outcomes in the domain of orthodontics remain under-researched. To date, there are no standardized clinical protocols or dosage guidelines for the use of synthetic MMPs inhibitors in OTM. Therefore, while these agents hold theoretical feasibility potential, particularly in periodontal patients undergoing orthodontic treatment, their clinical application in orthodontics requires validation through well-designed preclinical and clinical studies.

## 6. Conclusions

This review emphasizes the pivotal function of MMPs in the process of PDL and bone remodelling during OTM. Notwithstanding the presence of certain inconsistencies within the extant literature, MMPs have been identified as central enzymes in the process of ECM turnover, with particular emphasis on collagen degradation under compression. In this context, MMP-1, -2, -3, -9, and -13 have been the most consistently referenced. Furthermore, active MMPs have been observed to regulate other molecules and enzymes, the function of which is already relatively well established in bone resorption. Under conditions of tensile force, the activity of MMPs is subject to regulation by TIMPs, which facilitate the shift in balance towards tissue regeneration, with effects being more apparent for MMP-1 and -2 and TIMP-1. The influence of MMPs on angiogenesis and post-treatment relapse is a subject of increasing interest in the scientific community. A more profound comprehension of the interplay between MMPs and TIMPs could lead to enhanced treatment outcomes and increased success rates. This enhanced predictability has the potential to reduce adverse effects such as root resorption, and to facilitate the development of targeted therapeutic strategies. Synthetic MMPs inhibitors, despite demonstrating encouraging results and having already been clinically validated in the treatment of periodontitis, currently lack evidence, standardized protocols, and specific safety data for OTM. Consequently, they should be regarded as theoretical tools and are not yet ready for clinical use in orthodontics.

## Figures and Tables

**Table 1:** Prometallopeptidases activated by matrix metallopeptidases (MMPs) involved in periodontal ligament (PDL) remodelling.

MMPs	Activation
MMP-1 Collagenase	Pro-MMP-9
MMP-2 Gelatinase	Pro-MMP-13
MMP-3 Stromelysin	Pro-MMP-1 Pro-MMP-9 Pro-MMP-13
MMP-9 Gelatinase	Pro-MMP-13
MMP-10 Stromelysin	Pro-MMP-1 Pro-MMP-8 Pro-MMP-9
MMP-13 Collagenase	Pro-MMP-9 Pro-MMP-13 (self-activating mechanism)
MMP-14 Membrane-type MMPs	Pro-MMP-2 Pro-MMP-8 Pro-MMP-13

**Table 2:** Comparison of *in vitro* and *in vivo* findings on key matrix metallopeptidases (MMPs) during orthodontic tooth movement (OTM).

MMPs		*In Vitro* Findings	Main Cell Sources	*In Vivo* Findings	References
Collagenases	MMP-1	C: ↑ in compressed fibroblasts and osteoblasts	Fibroblasts, osteoblasts	C: ↑ in GCF or not detected	[77,117,118,120,124,125,126,127,132,136,142,147,148,149,150]
		T: ↑/↓/↔ in stretched fibroblasts		T: ↑ in GCF	
	MMP-8	C: ↑/↔ in compressed fibroblasts; ↔ in compressed osteoblasts	Fibroblasts, osteoblasts	C: ↑/↔ in GCF (and saliva)	[119,126,130,138,139,141,142,143,144,147,149]
		T: ↑ in stretched fibroblasts			
	MMP-13	C: ↑/↔ in compressed fibroblasts; ↑ in compressed osteoblasts	Fibroblasts, osteoblasts	↑ in GCF on both sides	[23,121,126,129,132,133,137,140,141,147,153]
		T: ↑ in stretched fibroblasts and osteoblasts			
Gelatinases	MMP-2	C: ↑/↓ in compressed fibroblasts; ↑ in compressed osteoblasts	Fibroblasts, osteoblasts	↑ in GCF on both sides	[117,118,127,128,132,133,136,145,147,148,151,152]
		T: ↑/↔ in stretched fibroblasts; ↓ in stretched osteoblasts			
	MMP-9	C: ↔ in compressed fibroblasts	Fibroblasts, osteoblasts,	↑ in GCF (and saliva) on both sides	[117,126,137,139,145,146,147,149,152]
		T: ↔ in stretched fibroblasts or not detected; ↑ in stretched osteoblasts			
Stromelysins	MMP-3	C: ↑ in compressed fibroblasts and osteoblasts	Fibroblasts, osteoblasts	↑ in GCF on both sides	[124,131,137,147]
	MMP-10	C: ↑ in compressed fibroblasts	Fibroblasts	No consistent trend reported	[124,147]
Membrane-type MMPs	MMP-14	C: ↑ in compressed osteoblasts	Fibroblasts, osteoblasts	No consistent trend reported	[117,132]
		T: ↔ in stretched fibroblasts			
	MMP-16	C: ↔ in compressed fibroblasts	Fibroblasts	No consistent trend reported	[126]
Matrilysins	MMP-7	C: ↔ in compressed fibroblasts	Fibroblasts	No consistent trend reported	[126,147]
Other MMPs		T: ↑ in stretched fibroblasts	Fibroblasts	C: ↑ in saliva of extraction-group individuals T: ↑ in GCF	[122,147]
	MMP-12				


C—compressed cells/compression side; T—stretched cells/tension side; arrows indicate observed changes in expression or activity: ↑ increased; ↓ decreased; ↔ no change.

## Data Availability

No new data were created or analysed in this study. Data sharing is not applicable to this article.

## Supplementary Materials

The following supporting information can be downloaded at: https://www.mdpi.com/article/10.3390/ijms27010542/s1.

## Abbreviations

The following abbreviations are used in this manuscript:

BMPs	Bone morphogenetic proteins
BSPs	Bone sialoproteins
CGRP	Calcitonin gene-related peptide
COX-2	Cyclooxygenase-2
CTGF	Connective tissue growth factor
ECM	Extracellular matrix
FAK	Focal adhesion kinase
FGF	Fibroblast growth factor
GCF	Gingival crevicular fluid
HIF-1α	Hypoxia-induced transcription factor 1 alpha
IFN-γ	Gamma interferon
IGF-1	Insulin-like growth factor 1
ILs	Interleukins
M-CSF	Macrophage colony-stimulating factor
MMPs	Matrix metallopeptidases
NE	Norepinephrine
OPG	Osteoprotegerin
OPN	Osteopontin
OTM	Orthodontic tooth movement
PDGF	Platelet-derived growth factor
PDL	Periodontal ligament
PGE_2_	Prostaglandin E_2_
RANK	Receptor activator of nuclear factor kappa-B
RANKL	Receptor activator of nuclear factor kappa-B ligand
SP	Substance P
TGF-β	Transforming growth factor beta
TIMPs	Tissue inhibitors of matrix metallopeptidases
TNF-α	Tumour necrosis factor-alpha
VEGF	Vascular endothelial growth factor
VIP	Vasoactive intestinal polypeptide
