# Supplementary material for: MMPs at Work: Deciphering Their Role in the Cellular Mechanisms of Orthodontic Tooth Movement
Source: Int J Mol Sci. 2026 Jan 5;27(1):542. doi: 10.3390/ijms27010542 (PMC12787207; doi:10.3390/ijms27010542)
Supplement: Supplementary file 1 [file ijms-27-00542-s001.zip › Supplementary Table S1 (In Vitro Studies).pdf]

Supplementary Table S1. A comparative overview of the experimental parameters and key findings of the *in vitro* studies on matrix metallopeptidases (MMPs) during orthodontic tooth movement (OTM).

Study	Biological sample/ cell type	Force device and intensity (compression/tension)	Sample time point(s)	Measured parameters	Key findings
**Compression studies**					
[77]	Human PDL fibroblasts from premolars	Centrifugal force: 36.3 g/cm² at 118 g	RNA harvested 24 h after onset of force for continuous and interrupted forces	mRNA levels of type I collagen (Col-I), MMP-1, and TIMP-1 by semi-quantitative RT-PCR.	Continuous force:<br>MMP-1 and Col-I ↑<br>TIMP-1 unchanged.<br><br>Interrupted forces:<br>MMP-1, Col-I, TIMP-1 unchanged
[125]	Human PDL fibroblasts from healthy premolars	Compressive force:<br>Continuous centrifugation at 167 g, generating ~33.5 g/cm² pressure on the cell layer; non-centrifuged cultures as controls	10, 20, 30, 60, 90, and 120 min of continuous centrifugation	mRNA for MMP-1, Col-I, TIMP-1, TIMP-2, (RT-PCR); cell viability (Trypan blue)	Compression:<br>mRNA MMP-1 ↑ (transiently)<br>Col-I, TIMP-1, and TIMP-2 ↑ (small, transiently)
[126]	Human PDL fibroblasts from healthy sites	Compressive force:<br>Custom pneumatic pressure bioreactor applying cyclic hydrostatic compression: 150 psi (~1 MPa, ~10 atm) at 0.1 Hz for 3 h on two consecutive days (physiological-like masticatory compression); unstimulated controls	mRNA collected after the 3 h compression period of two consecutive days	MMP and TIMP mRNA panel (MMP-1, -2, -3, -7, -8, -9, -10, -11, -12, -13, -14, -15, -16; TIMP-1, -2); two 84-gene qPCR arrays for ECM/adhesion and inflammatory cytokines/receptors<br>IL-6 mRNA (qRT-PCR) and protein (ELISA)	Compression:<br>MMP-1 ↑<br>MMP-8 e -9 ↓<br>IL-6 mRNA ↓
[127]	Human PDL fibroblasts from premolars and third molars	Custom tension–compression device applying 10% cyclic equibiaxial tension or 10% cyclic equibiaxial compression (release of 10% pre-stretch) at 30 cycles/min for 24 h; unstretched cultures as controls	After 24 h cyclic loading for tension, compression, control	MMP-2 and TIMP-2 mRNA (Northern blot); latent and active MMP-2 in supernatants (zymography and Western blot)<br>COL1A1 mRNA (qPCR);<br>Secreted COL-1 and fibronectin (ELISA, normalized per cell).	Compression:<br>mRNA and latent and active MMP-2 ↑<br>TIMP-2 unchanged<br>COL-1 and FN ↓<br>mRNA COL1A1 ↓<br><br>Tension:<br>mRNA MMP-2 and TIMP-2 ↑<br>COL-1 and FN ↑<br>mRNA COL1A1 ↑<br>total Protein ↑

Study	Biological sample/ cell type	Force device and intensity (compression/tension)	Sample time point(s)	Measured parameters	Key findings
[128]	Human PDL fibroblasts from third molars	Compressive force: Centrifugation at 141×g for 30, 60, 90, or 120 min (≈30.16 g/cm², ~290 g total load); non-centrifuged cultures as controls	Medium and cell lysates collected at 24, 48, and 72 h after centrifugation	MMP-2 and MMP-9 by gelatine zymography; MMP-3, MMP-7, MMP-10 by casein zymography.<br>total protein in medium and lysates (BCA)	MMP-3,-7, -9 and -10 not detected<br>MMP-2 activity<br>Secreted pro-MMP-2 ↓ at 24h (duration-dependent) and return to baseline at 72 h
[129]	Human PDF fibroblasts from premolars	Compressive force: Static continuous compression using weights of 2 g/cm² for 24 h, no weight as control	24 h of compression	mRNA for MMP-13, COX-2, IL-6, IL-8, IGF-1, VEGF (RT-qPCR); COX-2 and IL-8, PGE_2 protein (Western blot)/ELISA	MMP-13, COX-2, IL-8, IGF-1 ↑
[130]	Human PDL fibroblasts (HPdLF) and human osteoblasts (HOB-c)	Compressive force: Flexercell FX-3000™ applying static compressive deformation of 5% (~2 cN/mm², moderate) or 10% (~4 cN/mm², high) for 12 h; uncompressed cultures as controls	After 12 h compression for all groups	MMP-8, TIMP-1 e OPN protein in supernatants (ELISA);<br>Cell viability (MTT) and apoptosis (TUNEL);<br>mRNA from ALP, OCN, OPG and RANKL (qR-PCR)	Compression (5%):<br>HPdLF MMP-8 ↑<br>force-dependent TIMP-1 ↓ (high MMP-8/TIMP-1)<br>Both cell types ALP ↑<br>RANKL/OPG ↑<br>OPN strongly in HOB-c ↑<br><br>Compression (10%):<br>HOB-c viability ↓
[131]	Human osteoblast-like MG-63 cells and murine osteoblast MC3T3-E1 cells.	Compressive force: Flexercell™ applying cyclic compressive deformation: 1% and 5% (5.3 kPa, 10 s stretch/10 min relax); uncompressed cultures as controls.	Intermittently for up to 24 h	mRNA for MMP-3 (RT-PCR)	mRNA MMP-3 ↑ via a p38-MAPK–dependent signaling in both cell types
[132]	Human osteoblasts (Saos-2)	Compressive force: Static continuous compression using weights of 0.5, 1.0, 2.0, or 3.0 g/cm² for up to 24 h; no weight as control	1, 3, 6, 9, 12, and 24 h compression	mRNA (real-time PCR) and protein (ELISA) for MMP-1, MMP-2, MMP-3, MMP-13, MMP-14; TIMP-1, TIMP-2, TIMP-3, TIMP-4; tPA, uPA, and PAI-1	Compression at 1.0 g/cm² (defined as optimal):<br>MMP-1, MMP-2, MMP-14, and TIMP-1 ↑<br><br>Compression at 3.0 g/cm² :<br>mRNA MMP-3, MMP-13, TIMP-2/-3/-4, and tPA/uPA/PAI-1 ↑
[133]	Primary mouse calvaria osteoblasts cultured in 3D collagen-rich membranes	Compressive force: Flexercell™ applying dynamic compression: 6–10% deformation (1.0–1.67 MPa) at 1 Hz (sinusoidal) for 1–16 h; uncompressed 3D membranes as controls	1–16 h of cyclic compression	MMP-3, IL-6, PGE_2 (ELISA)<br>mRNA MMP-2, MMP-3, MMP-13 COLs, COXs and others	MMP-2, MMP-3, MMP-13 ↑<br><br>IL-6, COX-2, PGE_2 ↑<br>OPG, OPG/RANKL ↓ (10% > 6%, depended on α5β1 integrin–Ca^{2+}–ERK–NF-κB)

Study	Biological sample/ cell type	Force device and intensity (compression/tension)	Sample time point(s)	Measured parameters	Key findings
**Tension studies**					
[23]	Human PDL fibroblasts from healthy premolars	Tensile force:<br>Custom device using a convex-template strain method with applied lead weight resulting in continuous tension equibiaxial (~2.5% elongation for 0.25–6 h; unstrained cells as controls	15 min to 6 h of stretch,<br>30 min time point for gene expression arrays	RT² Profiler arrays for MAPK-related, ECM/integrin, growth factor, collagen, MMP, cytokine and angiogenic genes<br>Integrin β1/β3 (IIF)<br>FAK protein, p42/44 ERK and p38 total and phospho-forms; activated MMP-13 and TIMP-1 protein (WB)	MMP-12, MMP-13, OPN, ITGA3 ↑<br>TIMP-1 no change<br><br>MMP-10, COL1A1, COL14A1, VEGFA, CXCL1 ↓<br><br>Integrin β1/β3, FAK, ERK, p38 ↑<br>MAPK kinases and several BMP/FGFs ↑
[117]	Human PDL fibroblasts from healthy premolar.<br>Human gingival fibroblasts from healthy gingiva	Tensile force:<br>Flexercell ™ applying static tensile strain 20 kPa<br>Unstretched cells served as controls.	12 h	mRNA for MMP-1, MMP-2, MMP-9, MMP-14, TIMP-1, TIMP-2, TIMP-3 and α and β integrins (RT-PCR)	For both cell types:<br>MMP-1, MMP-2, TIMP-1, TIMP-2 ↑<br>MMP-9 not detected<br>MMP-14, TIMP-3 unchanged<br>Different pattern of integrins subunits expressiom
[119]	Human PDL fibroblasts (HPdLF)	Tensile force:<br>Flexercell FX-3000™ applying static tensile strain of 1%, 5%, or 10% elongation corresponding to 0.7, 3, and 5.2 cN/mm² for 12 h; unstretched cultures as controls	After 12 h	Secreted MMP-8, TIMP-1, IL-6, PGE_2 by ELISA<br>RANKL protein by immunofluorescence; IL-6 and COX-2 mRNA (qPCR)	Tensile strain (10%):<br>MMP-8 and TIMP-1 ↑<br>IL-6, COX-2, PGE_2 ↑<br><br>Tensile strain (5%):<br>TIMP-1 ↑<br>TIMP-1/MMP-8 ↑<br><br>Tensile strain (1%):<br>IL-6 ↓
[120]	Human PDL fibroblasts from healthy donors	Tensile force:<br>Flexercell FX-4000™ applying static equiaxial tensile strain of 1.5% for 1 h	At various times 0 –12 h	mRNA for MMP-1 and COL-I (qPCR); ERK, JNK, and p38 MAPK by immunometric assay kits	MMP-1 and COL-I ↑ controlled by ERK/JNK-AP-1 and ERK-NF- kB signaling pathways
[121]	Mouse osteoblasts MC3T3-E1	Tensile force:<br>Flexercell FX-3000™ applying tensile strain of 6%, 12%, or 18% elongation at 6 cycles/min for 24 h; unstretched cultures as controls	24 hours	MMP-13 and TIMP-1 mRNA (RT-PCR) and protein by western blot and ELISA	MMP-13 and TIMP-1 ↑ (mRNA and protein) with increasing magnitudes
[122]	Human PDL fibroblasts HPL	Tensile force:	24 hours	MMP-12, TIMP-1, TIMP-2, TIMP-3 mRNA (RT-PCR)<br>MMP-12 protein (ELISA, western blot)	MMP-12 ↑ (mRNA and protein)<br>$TIMP_S$ unchanged

Study	Biological sample/ cell type	Force device and intensity (compression/tension)	Sample time point(s)	Measured parameters	Key findings
		Cell Extender™ device applying tensile strain of 15% elongation: unstretched cultures as controls			
[148]	Human PDL fibroblasts from premolars	Tensile force:<br>Flexercell™ applying intermittent cyclic tensile strain: 20% elongation, 10 cycles/min (3 s stretch / 3 s relax) for up to 48 h	Up to 48 h	mRNA and protein for MMP-1, MMP-2, TIMP-1, TIMP-2, OPG and RANKL (qRT-PCT);<br>PGE_2 (EIA) levels; OPG (ELISA)	Intermittent tensile force:<br>MMP-1, MMP-2, and RANKL no change<br>TIMP-1 -2, OPG and PGE_2 ↑
[149]	Human PDL fibroblasts cells from premolar	Tensile force:<br>Flexercell FX-4000™ applying uniaxial cyclic tensile strain of 12% deformation for 5 s at 0.2 Hz every 90 s for 6, 12, or 24 h unstretched cultures as controls	6, 12, and 24 h of cyclic strain	RT² Profiler PCR arrays for 84 adhesion/ECM genes;<br>Cell viability (MTT) and apoptose (caspase-3/7)	MMP-8, MMP-11 and MMP-15 ↑<br>TIMP-1 and TIMP-2 (high mRNA but not responsive)<br>ADAMTS-1, COL6A1, COL8A1, COL11A1) ↓<br>Osteopontin, CTGF ↑<br>viability and apoptosis ↓ (small)<br>ITGA3, ITGA6, ITGA8, ITGB1<br>NCAM1 ↓
[150]	Human osteoblast-like PDL cells from third molars	Tensile force:<br>Flexercell™ -type system applying equibiaxial cyclic tensile strain of 1.8 to 12.5% elongation at 0.005 Hz with or not IL-1β,<br>unstretched cultures as controls		MMP-1, MMP-3, TIMP-1, -2, Osteocalcin, COX-2 and iNOS mRNA (RT-PCR)<br>PGE_2 (radioimmunoassay)<br>MMPs and TIMPs (western blot)	Observed effects only with IL-1β<br>Tensile strain (~3–6%): Antagonist of IL-1β<br>IL-1β-induced MMP-1 and MMP-3 mRNA and protein ↓<br>IL-1β-induced PGE_2 and COX-2, ↓<br>IL-1β-supressed TIMP-2 protein ↑<br>IL-1β-supressed osteocalcin ↑<br><br>Tensile strain (~10–12.5%):<br>Increase Inflammatory status
[151]	Human PDL fibroblasts from premolars	Tensile force:<br>Flexercell FX-4000™ applying cyclic biaxial tensile strain: 3% or 10% elongation at 0.1 Hz (5 s stretch/5 s relax) for 24 or 48 h;<br>unstretched cultures as controls	24 h and 48 h for mRNA, protein, and activity assays	mRNA for MMP-2, TIMP-2, COL1A1, COL3A1 and LOX (RT-PCR);<br>pro-MMP-2 and active MMP-2 by gelatin zymography.<br>LOX protein (western blotting) and enzymatic activity (fluorometric assay)	Low-level strain (3%):<br>mRNA MMP-2 or TIMP-2 unchanged<br>COL1A1, COL3A1 and LOX ↑<br>Total collagen ↑<br><br>10% elongation:<br>mRNA MMP-2 and TIMP-2 ↑<br>pro-MMP-2 ↑<br>COL1A1 ↓ COL3A1 ↑<br>Total collagen unchanged

Study	Biological sample/ cell type	Force device and intensity (compression/tension)	Sample time point(s)	Measured parameters	Key findings
[152]	Mousse osteoblast-like cells	Tensile force: Flexercell™ device applying cyclic tensile strain: 24% elongation at 0.05 Hz (10 s stretch/10 s relax) 3 cycles; unstretched cultures as controls	Continuously to periods of 0.5 h to 7 days	mRNA for MMP-2, MMP-3, MMP-9, MMP-10 (RT-PCR); MMP-2 and MMP-9 activity by gelatin zymography.	mRNA MMP-9 ↑, active MMP-9 unchanged<br>mRNA MMP-2, -3,-10 unchanged<br>active MMP-2 ↓

Arrows indicate observed changes in expression or activity: ↑ = increased; ↓ = decreased.
