# Supplementary material for: MMPs at Work: Deciphering Their Role in the Cellular Mechanisms of Orthodontic Tooth Movement
Source: Int J Mol Sci. 2026 Jan 5;27(1):542. doi: 10.3390/ijms27010542 (PMC12787207; doi:10.3390/ijms27010542)
Supplement: Supplementary file 1 [file ijms-27-00542-s001.zip › Supplementary Table S2 (In Vivo Studies).pdf]

Supplementary Table S2. A comparative overview of the experimental parameters and key findings of the human *in vivo* studies on matrix metallopeptidases (MMPs) during orthodontic tooth movement (OTM).

Study	Biological sample	Force device and intensity (compression/tension)	Sample time point(s)	Measured parameters	Key findings
[118]	GCF from tension and compression sides of left maxillary canines under initial fixed appliance loading and from controls	Fixed orthodontic MBT appliance with force application through an arch wire. Bands on first molars, 0.016-inch NiTi arch wire; teeth from left lateral incisor to contralateral first molar laced with 0.010-inch stainless steel wire. 150 g alloy coil spring for distal retraction of the left maxillary canine	GCF sampled at 1, 2, 3, 4 and 8 h after force application	MMP-1 (53 kDa) and MMP-2 (72 kDa) and related proteolytic activity	On the compression side: MMP-1 ↑ 1h, persists ≤3 h and return to baseline MMP2 ↑ till 8 h<br><br>On the tension side: MMP-1 ↑ 1h, persists ≤2 h and return to baseline MMP-2 ↑ till 1h and return to baseline
[136]	Saliva	Fixed orthodontic appliances: conventional brackets and self-ligating brackets 0.022-inch	Baseline, at 24 minutes and 1 month	MMP-1, MMP-2, leptin, fibronectin (FN) (ELISA)	MMP-1, FN ↓ MMP-2, leptin ↑
[137]	GCF from tension and compression sides of maxillary canines under initial fixed appliance loading and from controls	Fixed orthodontic appliance with force application through an arch wire. Continuous force during canine retraction	Follow-up over 80 days, sampled 7 days before activation, at activation, and after 1 h, 24 h, 14 days, 21 days and 80 days.	MMP-3, MMP-9, MMP-13; chemokines MIP-1β, MCP-1, and RANTES assessed by multiplex bead-based immunoassay	On the compression side MMPs ↑ 1h MMPs ↓ 24h MMPs ↑ thereafter No changes on tension side<br><br>No changes for the chemokines
[138]	GCF from teeth under initial fixed appliance loading and from controls	Fixed orthodontic appliance with force application through an arch wire. Continuous force	Sampled hourly for the first 8 hours after activation.	MMP-8 (IFMA) MMP-1 and MMP-8 (Western blot)	MMP-8 ↑ just the minor part is active MMP-1 not detected
[139]	Saliva and GCF	Fixed orthodontic appliance with force application through an arch wire. Constant force	Baseline and at 1, 3, 6, 9 and 12 months during treatment	MMP-8, MMP-9, IL-1β, TNF-α, and RANKL/OPG ratio (ELISA)	MMP-8, -9 and RANKL/OPG ↑ (1st 3 months) IL-1β and TNF- α ↑ (1st month)
[142]	GCF from teeth under fixed appliance loading and from controls	Fixed orthodontic appliance with force application through an arch wire. Continuous force	Follow-up over 1 month with sampling every 24 hours	MMP-8 (IFMA) Molecular forms of MMP-1 and MMP-8 (Western blot)	MMP-8 ↑, just the minor part is active. MMP-1 not detected
[143]	GCF from a single “representative” tooth	Fixed orthodontic appliance with force application through an arch wire	Follow-up over 3 months; baseline, 24 h, 1 week and 3 months	MMP-8 (ELISA)	No changes during the first week MMP-8 ↑ at 3-months

Study	Biological sample	Force device and intensity (compression/tension)	Sample time point(s)	Measured parameters	Key findings
[144]	GCF	Orthodontic clear aligners (Invisalign Teen) worn ~22 h/day	Baseline and at 4 weeks, 6, 12, 18 months and 1 year after treatment	Active MMP-8 (ELISA), 11 periodontitis-associated marker bacteria bacterial (LCD Array Kit BAC-Dent 2.4), IL-1 polymorphism	Active MMP-8 unchanged orange and green bacterial complexes ↑
[145]	GCF samples were collected from all teeth in the 2nd segment on the buccal and lingual sides, and then pooled	Fixed orthodontic appliance: 0,022-inch self-ligating brackets with 0.014-inch beta-Ti arch wire	Follow-up over first 6 weeks; samples were taken every first 4 weeks and the 6th week	pro-MMP-9, multimer MMP-9, and MMP-2 complex (Gelatin zymography); total protein (BCA assay)	MMPs unchanged and not correlate with OTM rate
[146]	GCF from tension and pressure sides from test and controls teeth	Fixed orthodontic MBT appliance with force application through a 0.014-inch NiTi, 0,018-inch NiTi and a 0.018-inch SS arch wire sequence. 100 g NiTi closing coil applied between maxillary canine and first molar. Routine clinical activation	Follow-up over 42 days. Sampling at baseline, 4 hours, 7 days and 42 days	MMP-9, TIMP-1/2, IL-1β, IL-6, IL-8, TNF-α, GM-CSF), RANKL and OPG (Multiplex assay, Luminex)	On the compression side MMP-9 ↑ 7 and 42 days IL-1β and IL-8 ↑ 4h RANKL ↑ 42 days<br><br>On tension sides: MMP-9 and TIMPs 1 and 2 ↑ across all time points IL-1β, IL-8, TNFα ↑ across all time points
[147]	Saliva	Fixed orthodontic appliance with 0.018-inch bracket slot. A 0.014-inch NiTi arch-wire was placed for the initial leveling and alignment stage. No further appliance reactivations during sampling time	One week before, at 0h, 1h and 8 weeks	MMP-1, MMP-2, MMP-3, MMP-7, MMP-8, MMP-9, MMP-10, MMP-12, and MMP-13 (Multiplex assay, Luminex)	MMP-8 and MMP-9↑ at 1h, return to baseline at 8 h<br><br>MMP-1, MMP-3, MMP-7, MMP-10, MMP-12, MMP-13 not altered<br><br>MMP-2 not detected

Arrows indicate observed changes in expression or activity: ↑ = increased; ↓ = decreased.
